# Efficacy of melatonin in animal models of intracerebral hemorrhage: a systematic review and meta-analysis

**DOI:** 10.18632/aging.202457

**Published:** 2021-01-27

**Authors:** Liuwang Zeng, Yuwei Zhu, Xiangyu Hu, Haiyun Qin, Jiayu Tang, Zhiping Hu, Chunli Chen

**Affiliations:** 1 Department of Neurology, Second Xiangya Hospital, Central South University, Changsha 410011, Hunan, China; 2 Department of Neurology, The Second People’s Hospital of Hunan Province, Changsha 410007, Hunan, China

**Keywords:** melatonin, ICH, systematic review, meta-analysis, animal models

## Abstract

Melatonin is a potent antioxidant and anti-inflammatory agent that is showing promising results in acute brain injury. The aim of this study was to systematically evaluate the pre-clinical evidence on the effectiveness of melatonin in improving outcome after intracerebral hemorrhage (ICH). We searched mainstream databases from the inception to the end of June 2020. Outcomes were measured by neurobehavioral scores or brain water content. Meta-analyses were performed with Stata 12.0 and Review Manager 5.3. Finally, 8 articles published from 2008 to 2019 met the inclusion criteria. Meta-analysis of pre-clinical data revealed an overall positive effect on neurobehavioral outcome with a standardized mean difference (SMD) of -0.81 (95% CI: -1.47, -0.15; p = 0.016) with significant heterogeneity (Q = 41.49, I^2^ = 68.7%; p = 0.000). Further subgroup analysis were performed from methodological differences, especially dose and timing of treatments. Furthermore, melatonin reduced cerebral edema by an SMD of -0.78 (95% CI: -1.23, -0.34; p = 0.001) with low heterogeneity. In conclusion, melatonin treatment significantly improves both behavioral and pathological outcomes in animal models of ICH. In addition, the results should be interpreted in light of the limitations in experimental design and methodological quality of the studies included in the meta-analysis.

## INTRODUCTION

Intracerebral hemorrhage (ICH) is one of most fatal subtypes of stroke caused by rupture of blood vessels in brain parenchyma, and has extremely high morbidity and mortality worldwide [1, 2]. The mortality rate of acute ICH is approximately 40% in the first three weeks, and those who survive often suffer from different degrees of neurological deficit [3]. Currently, management of ICH is largely carried out via mechanically removing the hematoma, decreasing intracranial pressure, controlling severe brain edema and maintaining life function [4]. However, these treatments are not yet sufficiently effective to improve ICH survival rates and promote functional recovery [5, 6]. Therefore, new treatment drugs need to be explored under the guidance of evidence-based medicine.

Melatonin (N-acetyl-5-methoxytryptamine) is a type of pineal gland hormone, which is secreted from the pineal gland during the dark phase of the light–dark cycle [7, 8]. It has a variety of biological properties: scavenging reactive oxygen species (ROS), increasing antioxidant enzymes, protecting mitochondrial function, reducing inflammation, and inhibiting apoptosis [9–12]. Abundant experimental evidence has demonstrated that melatonin could be a promising neuroprotective agent in both acute brain injuries and chronic neurodegenerative diseases [13–15]. In particular, the neuroprotective effects of melatonin have been repeatedly tested in different experimental models of ICH [16]. For example, Rogas et al. [17] found that melatonin (15 or 150mg/kg) administration did not improve neurobehavioral outcome or brain edema at 24 h post-ICH. Moreover, Lekic et al. [18] demonstrated that melatonin (5 or 15mg/kg) failed to show any significant effect on brain edema and neurological deficits at one day post ICH, in spite of oxidative stress reductions. On the contrary, positive results of melatonin administration in animal models of ICH have also been reported, including improvements in neurological outcome as well as reductions in brain water content [19–21]. To date, there is no systematic review and meta-analysis to evaluate the quality of pre-clinical studies and synthesize evidence on the effects of melatonin with ICH. The aim of this study is to provide evidences relating to the efficacy of melatonin treatments on the behavioral and pathological outcome in animal models of ICH, to inform and guide the design of evidence-based, large-scale clinical trials, and supporting the clinical application of melatonin administration against ICH.

## RESULTS

### Study selection

**Figure 1:**
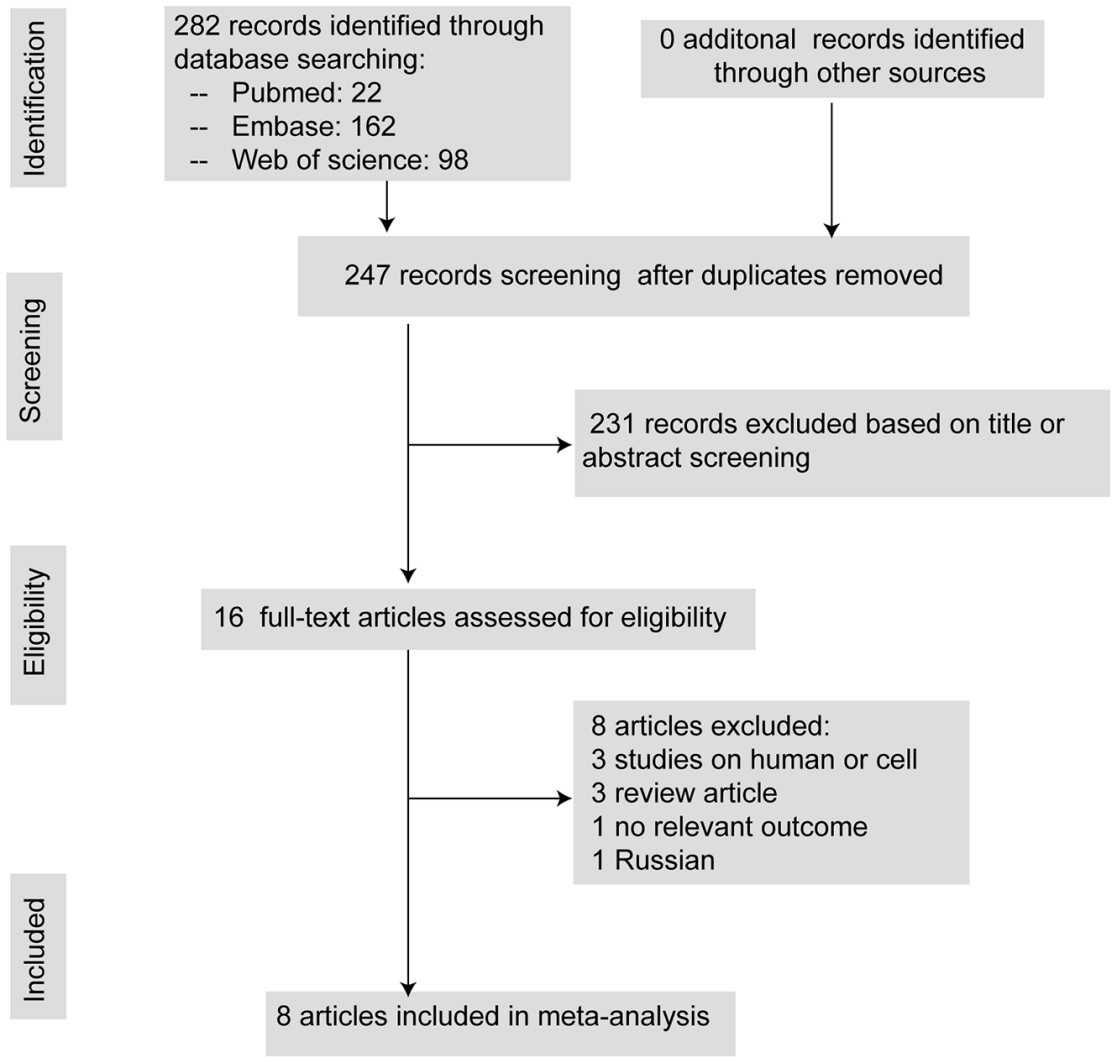
**The flow diagram describing literature search and study selection.**

The systematic review and meta-analysis were conducted and reported in compliance with Preferred Reporting Items for Systematic Review and Meta- Analyses (PRISMA) guidelines [22]. The literature search identified 282 potential studies, at the primary retrieval. After review and exclusion, 16 full-text articles remained, which were then assessed for inclusion eligibility. From these, 8 records were excluded due to the reasons given in Figure 1. Finally, this systematic review included eight articles published from 2008 to 2019 that met the inclusion criteria, which comprised 15 comparisons describing the neurobehavioral scores and 14 comparisons describing the brain water content.

### Study characteristics

The overall study characteristics are shown in Table 1. The eight included studies involved Sprague–Dawley rats (n = 5) [17–20, 23], Wistar rats (n = 2) [24, 25], and C57 mice (n = 1) [21]. Most studies used male animals (n = 7), while one publication [23] did not mention the gender of animals and used neonatal animals. Half of the studies used autologous blood model (n = 4) and the remaining four studies applied collagenase model [17, 18, 23, 25]. All interventions were given by intraperitoneal (IP) injection except in one study, in which it was administered orally [25]. The initial dosage of melatonin ranged from 5mg/kg to 150mg/kg, although the most frequent dose was 15mg/kg. The intervention was given once in 11 unique comparisons of five studies [17, 18, 20, 21, 23]. and the dose was repeated or multiple times in 4 unique comparisons of three studies [19, 24, 25]. The total melatonin dose ranged from 5 mg/kg to150 mg/kg. The interventions of different comparisons were commenced 30 min or 1 h before the ICH induction [21, 25] or 15 min [17, 18] or 1 h [19, 20, 23, 24]. or 3 h [17, 18] or 6 h post ICH [25]. Assessment was performed 24 h to 4 weeks after induction of ICH.

**Table 1:** Characteristics of comparisons which included in 8 elected studies.

**Author, Year**	**Animal, gender**	**Age**	**Anesthetic drugs**	**Method of ICH**	**Initial dose**	**Total dose**	**Treatment point**	**Route**	**Treated(n)/ control(n)**	**Assessment time**	**Outcome measure (direction)**
Rogas, 2008	SD rats, Male	Adult	isoflurane	collagenase	15mg/kg	15mg/kg	15min post ICH	IP	4,4	24 h	BWC(lower is better^a^); Garcia score (higher is better^b^)
Rogas, 2008	SD rats, Male	Adult	isoflurane	collagenase	15mg/kg; 150mg/kg	15mg/kg; 150mg/kg	3h post ICH	IP	4,2; 4,2	24 h	BWC(lower is better); Garcia score (higher is better)
Li. 2009	Wistar rats, Male	Adult	chloral hydrate	Whole blood	10mg/kg	20mg/kg	1h, 4h post ICH	IP	5,5	24 h	BWC(lower is better)
Lekic, 2010	SD rats, Male	Adult	isoflurane	collagenase	15mg/kg	15mg/kg	15min post ICH	IP	6.6	24 h	BWC(lower is better); Garcia score (higher is better)
Lekic, 2010	SD rats, Male	Adult	isoflurane	collagenase	5mg/kg; 15mg/kg	5mg/kg; 15mg/kg	3h post ICH	IP	6,3 6,3	24 h	BWC(lower is better); Garcia score (higher is better)
Lekic, 2011	SD rats, NR	Neonatal	isoflurane	collagenase	5mg/kg; 10mg/kg	5mg/kg; 10mg/kg	1h post ICH	IP	8,4; 8,4	4 weeks	Neurodeficit score (lower is better)
Ueda, 2014	Wistar rats, Male	Adult	pentobarbital	collagenase	15mg/kg	105mg/kg	every 24h for 7d (from 6h post ICH)	Oral	14,12	1d, 3d, 7d	Motor deficit score (lower is better)
Ueda, 2014	Wistar rats, Male	Adult	pentobarbital	collagenase	15mg/kg	105mg/kg	every 24h for 7d (from 1h before ICH)	Oral	9,19	1d, 3d, 7d	Motor deficit score (lower is better)
Wang, 2018	SD rats, Male	Adult	Chloral hydrate	Whole blood	5mg/kg	15mg/kg	1h,24h,48h post ICH	IP	12,12	72h	BWC(lower is better); Clinical behavioral scores (lower is better)
Xu, 2018	SD rats, Male	Adult	pentobarbital	Whole blood	100mg/kg 150mg/kg	100mg/kg; 150mg/kg	1h post ICH	IP	6,3 6,3	24h	BWC(lower is better); NSS score (higher is better)
Lu, 2019	C57 mice, Male	Adult	pentobarbital	Whole blood	20mg/kg	20mg/kg	30min before ICH	IP	6,6	1d,3d,7d	BWC(lower is better); Neurodeficit score (lower is better)

Legends: lower is better^a^: = lower value indicates more favorable outcome; higher is better^b^: = higher value indicates more favorable outcome; Con, control; SD, Sprague–Dawley; NR, not reported; IP: intraperitoneal injection; BWC, brain water content.

### Study quality

The quality score of the studies ranged from 4 to 7 (mean 5.5), among them 7 (87.5%) included studies were regarded as high methodological quality (≥5) studies. All studies have been published in peer-reviewed journals and stated compliance with animal welfare regulations. 6 of 8 studies reported describing control of temperature; 5 of 8 studies reported randomized allocation to treatment group; 3 of 8 studies reported blinded assessment of outcome. None of them used masked induction of haemorrhage or used animals with relevant comorbidities (e.g. hypertension) or reported a sample size calculation or used anesthetics with known marked intrinsic neuroprotective properties such as ketamine. 6 studies stated possible conflicts of interest. The details of quality index are concluded in Table 2.

**Table 2:** Methodological quality of 8 studies included in the meta-analysis.

**Study**	**(1)**	**(2)**	**(3)**	**(4)**	**(5)**	**(6)**	**(7)**	**(8)**	**(9)**	**(10)**	**Total**
Rogas, 2008	√	√			√	√			√		5
Li. 2009	√	√	√			√			√		5
Lekic, 2010	√	√	√		√	√			√	√	7
Lekic, 2011	√		√			√			√	√	5
Ueda, 2014	√					√			√	√	4
Wang, 2018	√	√	√		√	√			√	√	7
Xu, 2018	√	√				√			√	√	5
Lu, 2019	√	√	√			√			√	√	6

Legends: (1) peer reviewed publication; (2) control of temperature; (3) random allocation to treatment or control; (4) blinded induction of haemorrhage; (5) blinded assessment of outcome; (6) use of anesthetic without marked intrinsic neuroprotective activity; (7) animal model (aged, diabetic or hypertensive); (8) sample size calculation; (9) compliance with animal welfare regulations; and (10) statement of potential conflict of interests.

### Global estimates of efficacy

**Figure 2:**
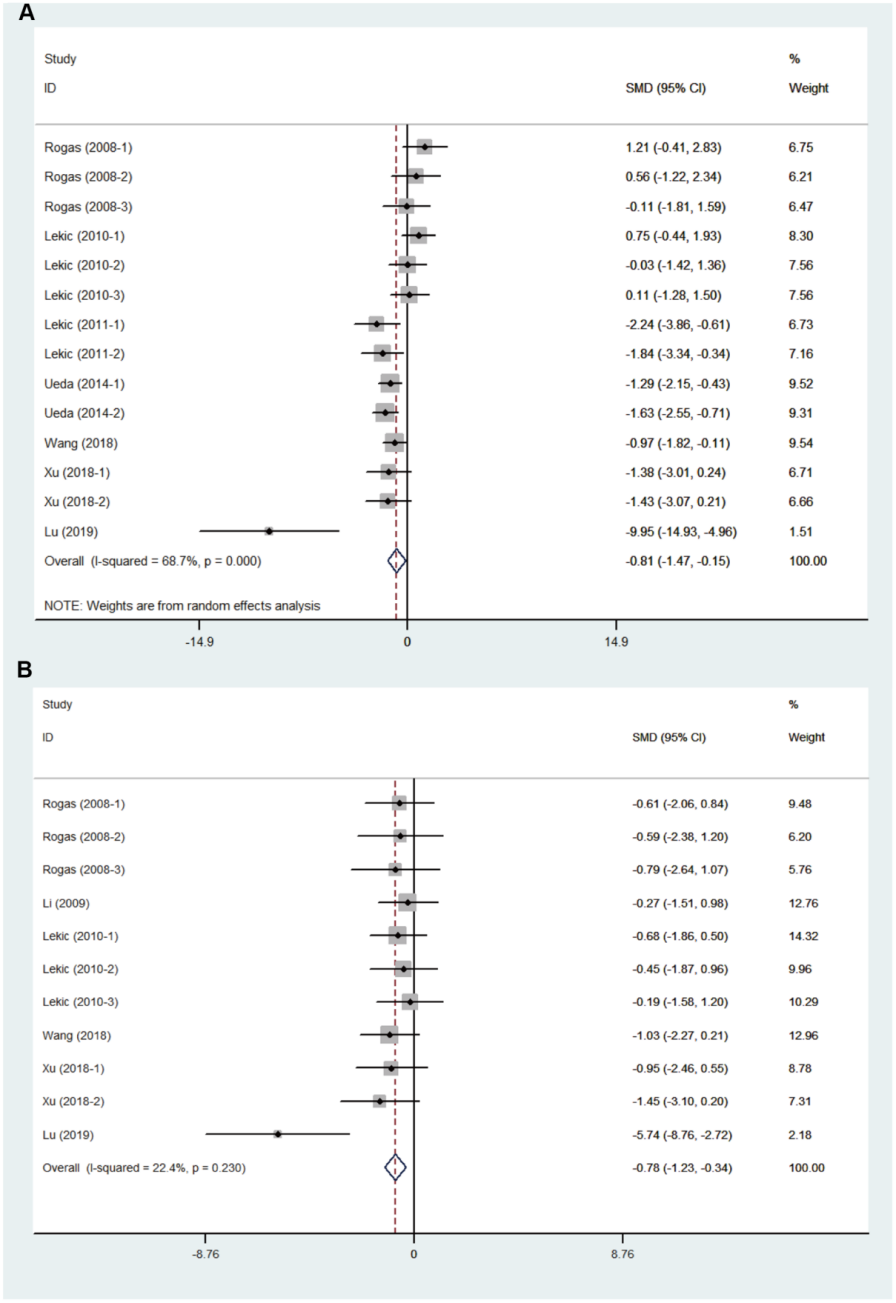
**Effect size of included comparisons.** Forest plot shows mean effect size and 95 % CI for (**A**) neurobehavioral outcomes and (**B**) brain water content.

Melatonin treatment had a favorable effect on the neurobehavioral outcome (Figure 2A), by an SMD of -0.81 (95% CI: -1.47 to -0.15; p=0.016, seven studies, 14 comparisons, Figure 2A). The heterogeneity among comparisons of neurobehavioral outcomes was statistically significant (Q= 41.49, I^2^ = 68.7%; df = 13; p=0.000), so we did further subgroup analysis from methodological differences, especially dose and timing of treatments. On the other hand, melatonin reduced the brain water content by an SMD of -0.78 (95% CI: -1.23, -0.34; p=0.001, 7 studies, 11 comparisons, Figure 2B). The heterogeneity among comparisons of brain water content was low (Q = 12.88, I^2^ = 22.4%, df = 10, p =0.23); therefore, further subgroup analysis was not performed.

### Sensitivity analysis

**Figure 3:**
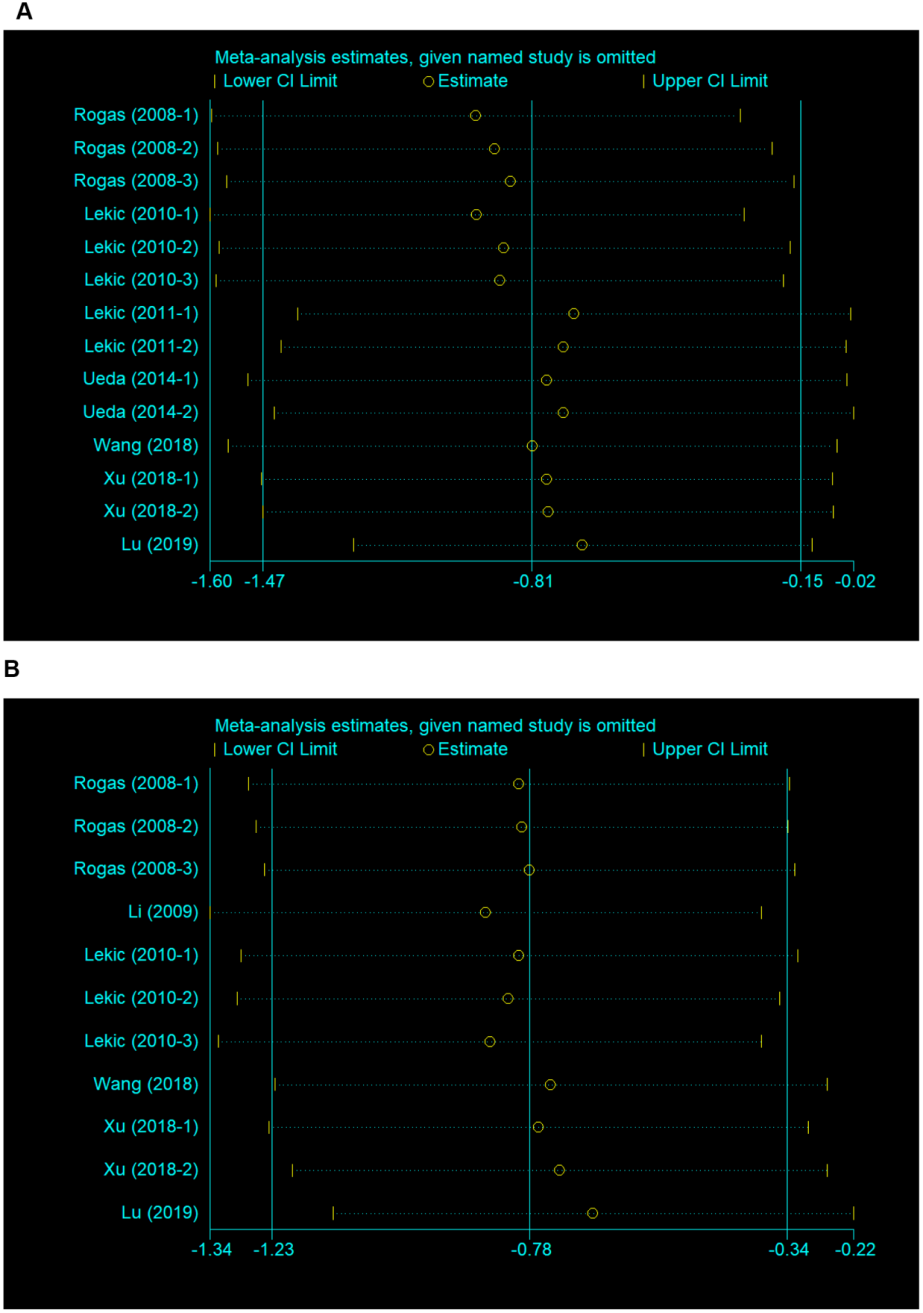
**Sensitivity analysis for the included comparisons.** Figures show mean effect size and 95 % CI for (**A**) neurobehavioral outcomes and (**B**) brain water content.

We conducted a sensitivity analysis to evaluate the stability of the results by sequential omission of each study if heterogeneity between the studies existed. The pooled SMD of neurobehavioral outcome was not significantly affected by any study, nor was the brain water content (Figure 3A, 3B).

### Publication bias

**Figure 4:**
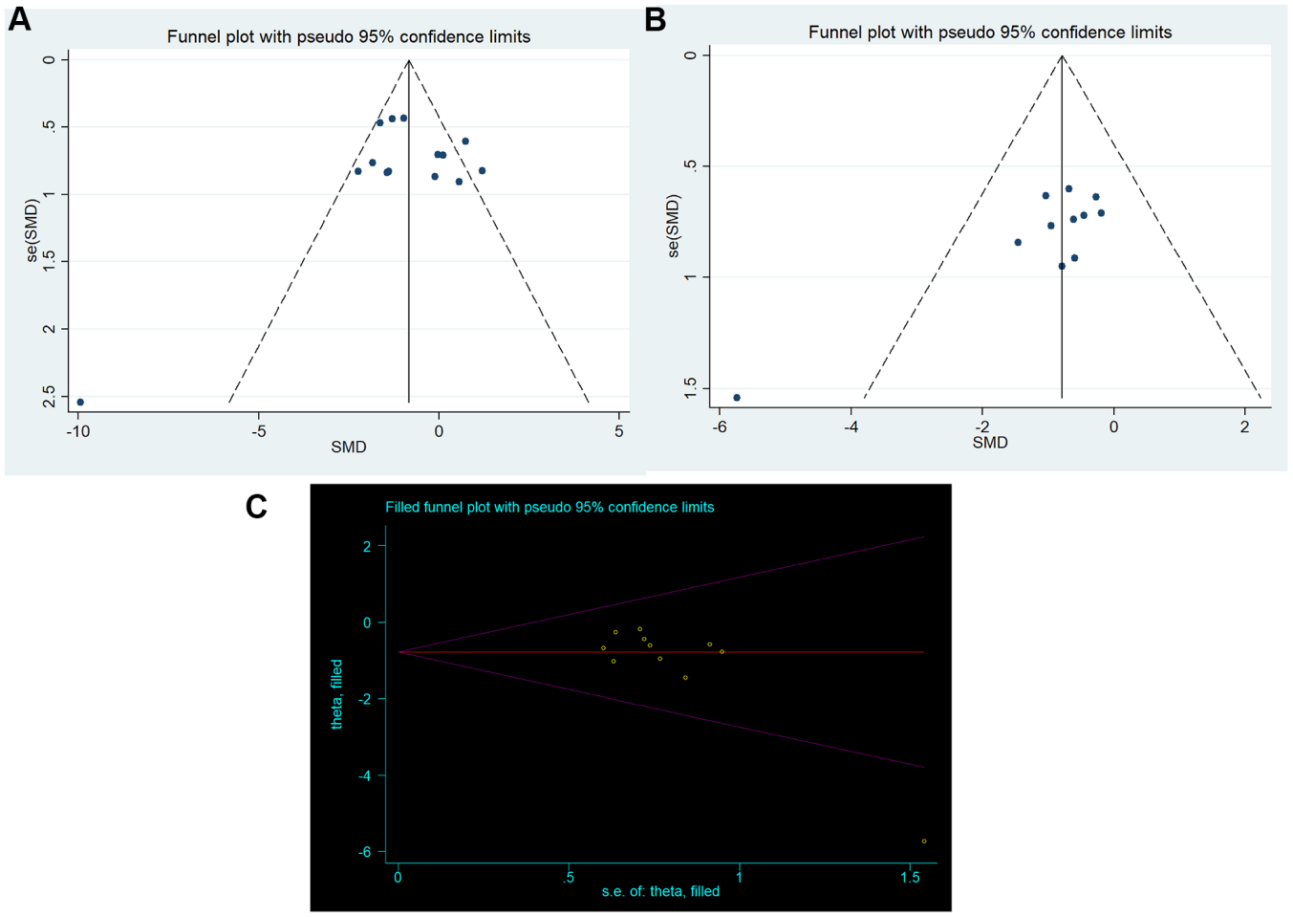
**Publication bias.** Funnel plots for (**A**) neurobehavioral outcomes and (**B**) brain water content; (**C**) trim-and-fill method.

The funnel plot was approximately symmetrical for the comparisons of neurobehavioral outcome (Figure 4A), and the results from the Egger’s test confirmed no significant publication bias (p=0.658).

For the comparisons of brain water content, the funnel plot showed some asymmetry, which may suggest the presence of publication bias (Figure 4B). The results from the Egger test confirmed significant bias (p=0.007). Then, we used the trim-and-fill method to estimate missing studies and recalculated the overall pooled effect estimates. The imputed effect estimates were consistent (SMD -0.784, 95% CI: -1.230 to -0.338, p =0.001), indicating no “missing” studies (Figure 4C).

### Subgroup analysis

Details of stratified analysis on neurobehavioral score are given in Table 3. If studies used neurobehavioral scales describing functional benefits, we classify them in “positive direction” group. On the contrary, if studies used neurobehavioral scales describing functional deficit, we classified them in “negative direction” group. We found significant differences in estimates of effect size between “positive direction” and “negative direction” groups (χ2 = 10.43, df = 1, p = 0.001, Supplementary Figure 1). Studies classified as low- and high-quality ones showed no significant difference in estimates of effect size (χ2 = 2.19, df = 1, p = 0.14, Supplementary Figure 2). The methods used to induce ICH model showed no significant differences in estimates of effect size (χ2 = 2.54, df = 1, p = 0.11, Supplementary Figure 3), nor was there any distinction among studies using different anesthetics drugs (χ2 = 5.50, df = 2, p = 0.06, Supplementary Figure 4). The methods of melatonin administration also showed no significant differences in estimates of effect size (χ2 = 2.19, df = 1, p = 0.14, Supplementary Figure 5). The melatonin dosage administered ranged from 5 mg/kg to 150 mg/kg, and the neuroprotective effects were not seen with all doses of melatonin (Supplementary Figure 6). Although our study demonstrated that the greatest effect was exerted at a dosage of 20mg/kg (Supplementary Figure 6), the wide dose range made the assessments less reliable. In addition, there was no significant difference between single dose and multiple dosage groups (χ2 = 1.24, df = 1, p = 0.27, Supplementary Figure 7). Finally, there was no significant difference between the pre-treatment and post-treatment groups (χ2 = 1.36, df = 1, p = 0.24, Supplementary Figure 8).

**Table 3:** Stratified meta-analysis of neurobehavioral score.

**Subgroup analysis**	**No. of studies**	**SMD (95%CI)**	**Heterogeneity test**			**χ2**	**p**
			**Q**	**I^2^**	**p_Q_**		
3.1 neurobehavioral scales							
positive direction	6	-1.74(-2.59,-0.89)	13.82	64%	0.02		
negative-direction	8	-0.01(-0.64,0.63)	9.76	28%	0.20		
						10.43	0.001*
3.2 Study quality							
high	12	-0.68 (-1.48, 0.11)	35.93	69%	0.000		
low	2	-1.45 (-2.08, -0.82)	0.28	0%	0.59		
						2.19	0.14
3.3 Methods to induce ICH							
collagenase	10	-0.51 (-1.23, 0.22)	27.05	67%	0.001		
whole blood	4	-2.03 (-3.76, -0.30)	12.18	75%	0.007		
						2.54	0.11
3.4 Anesthetics							
pentobarbital	5	-1.78 (-2.85, -0.71)	11.34	65%	0.02*		
isoflurane	8	-0.18 (-1.01, 0.64)	16.90	59%	0.02*		
chloral hydrate	1	-0.97 (-1.82, -0.11)	0.00				
						5.50	0.06
3.5 Routes of injection							
Intraperitoneal injection	12	-0.68 (-1.48, 0.11)	35.93	69%	<0.001*		
Oral injection	2	-1.45(-2.08, -0.82)	0.28	0%	0.59		
						2.19	0.14
3.6 Dose administration							
single dose	11	-0.68 (-1.60, 0.24)	34.83	71%	<0.001*		
multiple dosage	3	-1.28 (-1.78, -0.77)	1.08	0%	0.58		
						1.24	0.27
3.7 Time administration							
Pre-ICH treatment	2	-5.41 (-13.53, 2.7)	10.34	90%	0.001*		
Post-ICH treatment	12	-0.58 (-1.17, 0.01)	24.84	56%	0.01*		
						1.36	0.24

Legends: *means p < 0.05.

## DISCUSSION

### Summary of evidence

As a promising neuroprotective candidate, there is extensive and systematically summarized preclinical evidence on the use of melatonin and its potential to improve neurobehavioral and pathological outcomes in animal models with ischemic stroke [26] and traumatic brain injury [27]. However, the potential of melatonin in animal models of ICH remains unknown. To our knowledge, this is the first systematic review of the evidence evaluating the literature for the efficacy of melatonin in animal models with ICH. Our study demonstrated that melatonin significantly improved neurobehavioral outcome [SMD -0.81 (95% CI: -1.47, -0.15)] and reduced cerebral edema after ICH [SMD -0.78 (95% CI: -1.23, -0.34)]. Based on the results of our meta-analysis in pre-clinical studies, there is potential for treatment with melatonin to improve functional outcome with acute ICH.

### Possible mechanisms for the effect of melatonin in ICH

Although the role of melatonin in the chronoregulation of major physiological processes (e.g., the light–dark cycle) was well accepted, its therapeutic potential was only gradually explored in ICH. Ueda et al. demonstrated that oral administration (15 mg/kg) for 7 days after ICH resulted in significant recovery of motor function via reducing oxidative stress and enhancing electrical responsiveness [25]. Besides, other properties of melatonin have been described in ICH, including a neuroprotective effect in mitochondrial function, an inhibiting effect in proinflammatory cytokine production and anti-apoptotic effects [19]. In addition, Lu et al. found that pretreatment with melatonin suppressed necroptosis of microglia in mice via regulating the deubiquitinating enzyme A20 [21]. Thus, we propose that melatonin is a promising neuroprotective candidate that is worthy of further evaluation for its potential therapeutic application in this devastating disease.

### Interpretation of subgroup analysis

The heterogeneity among comparisons of neurobehavioral outcomes was statistically significant (χ2 = 41.49, I^2^ = 68.7%; df = 13; p=0.016), so we did further subgroup analysis for methodological differences, especially dose and timing treatments.

### Neurobehavioral scales

Significant differences in estimates of effect size were found between “positive direction” and “negative direction” groups. The reality is that there are diversified neurobehavioral scale for ICH animal models around the world [28], and the score of different scales were sometimes affected by observers with different experiences. Thus, developing a unified and standardized scale for assessing neurobehavioral score is very important to decrease the heterogeneity between individual studies.

### Study quality

There was no clear relationship between the score of quality and the estimate of effect size. Some analyses found grouping studies by quality may account for substantial heterogeneity between them [29, 30], but others found no significant difference in effect size among studies with different research design qualities [31, 32]. In our meta-analysis, low quality studies tended to show higher efficacy, but no significant difference was observed between low- and high- quality studies. Besides, to what extent deficiencies in study quality might have overestimated the effect of melatonin was not known. Therefore, this area remains to be explored in depth in future research.

### Methods of animal model induction

Bacterial collagenase model and autologous blood model are most commonly used in animal models of ICH. MacLellan et al. [33] reported that hematoma size became larger in the stage of secondary brain injury and severe neurological dysfunction occurred in the collagenase model rather than autologous blood model. Besides, bacterial collagenase model produced greater edema, and inflammation than autologous blood model [34]. Despite the differences of pathophysiological mechanisms in these two models, we found no significant difference in estimates of effect size between studies using autologous blood model and those using collagenase model. Therefore, both models can be used in the test for effective ICH therapies.

### Anesthetic

One systematic review found that phenobarbital anesthesia showed the most effective result for deferoxamine treatment in animal models of ICH [35]. However, our results showed no significant difference of effect size among the use of phenobarbital, isoflurane, and chloral hydrate. As commonly used anesthesia drugs, though they have demonstrated varying degrees of neuroprotective properties [36–38], their efficacy remains uncertain in the experimental ICH model [39]. Therefore, new anesthetics may need to be sought out with minimized influence on the pathophysiologic process of ICH.

### Time and dosage

The dose, timing, and frequency of interventions varied greatly, making it difficult to draw conclusions. Although our results showed that the greatest effect was exerted at dosage of 20 mg/kg, the wide dose range made the assessments less reliable. In humans, melatonin has an elimination half-life of approximately 45 minutes following oral or intravenous administration [40]. On the other hand, the elimination half-life of melatonin following oral or intravenous administration in rats is less than 20 min [41], so multiple-dose treatment of melatonin can overcome the short duration of action *in vivo* and show increased efficacy compared with single-dose treatment in ischemia stroke. However, in our study, the effect size of studies using multiple dosage was not significantly different from those using once-only treatment. These may be affected by various dosage and small sample size in individual studies. In addition, the time of melatonin treatment theoretically could target different mechanisms. For instance, early administration of melatonin can target glutamate toxicity and free radical formation [42]; and late treatment can target neuroinflammation [9]. As we know, secondary brain injury is a rapidly progresses after ICH [43], especially in the first few hours. Thus, pre-administration of melatonin may be more neuroprotective. However, in our study there was no difference of effect size between pre-treatment group and post-treatment group. Indeed, significant heterogeneity still existed in both subgroups. Therefore, the dosage and timing of melatonin administration should be standardized in future trials to minimize the degree of heterogeneity.

### Strengths and limitations

This study made great efforts to arrive at a relatively objective result. First, the study tried to collect most reports in this field, and therefore, provided the most complete evidences of melatonin in ICH animal models. Second, to reduce potential bias in assessing the methodological quality of the included studies, two practiced investigators independently evaluated and extracted data from all included studies. Finally, our results showed that melatonin treatment significantly improved both behavioral and pathological outcomes in animal models of ICH and sensitivity analysis confirmed stable results of neurobehavioral outcome as well as brain water content.

However, the present systematic review and meta-analysis has some limitations. First, although our search strategy was exhaustive, it is also possible that some published studies were missed. Second, the meta-analysis was limited by a small data set; although 282 publications were identified through electronic search, only 8 publications were found to meet our criteria. As a result, further studies with large sample sizes are warranted to provide sufficient evidence about the effect of melatonin on ICH. Third, in our meta-analysis, it was not possible to examine the effects of melatonin in specific ICH populations with comorbidities such as diabetes or hypertension, who may have different responses to melatonin treatment. Thus, there is significant work to be done when it comes to clinical translation.

## CONCLUSIONS

Melatonin is an old drug that has been investigated for many years, and therefore has an advantage over new drugs that are not well characterized. Our current meta-analysis demonstrates that melatonin treatment significantly improves both neurobehavioral and pathological outcomes in animal models of ICH. However, the results should be interpreted in light of the limitations in experimental design and methodological quality of the studies included in the meta-analysis. Therefore, further studies are warranted to improve study quality and reduce potentially confounded publication bias.

## MATERIALS AND METHODS

### Search strategy

We searched: PubMed, Embase and Web of Science from the inception to the end of June 2020. Medical Subject Headings and keywords related to melatonin and intracerebral hemorrhage were used in each database. Search strategy was provided in detail (Supplementary File 1).

### Inclusion and exclusion criteria

Studies were included if they fulfilled the following criteria: (1) experimental ICH was induced and the therapeutic effect of melatonin was assessed; (2) control animals were used (saline, or similar vehicle); (3) melatonin was administered before the injury or at any time-point post-injury; (4) effect of melatonin was assessed by neurobehavioral outcome or brain water content; (5) There had to be full text available within a peer-reviewed journal, published in English. Articles that reported on the same sample were treated as a single study. Two reviewers (Zeng, LW and Zhu, YW) independently screened the abstracts according to the inclusion criteria, and disagreements were addressed by discussion with a third reviewer (Xiangyu, Hu). The meta-analysis excluded studies that used non-traumatic models of hemorrhagic injury or individual comparisons from which we could not calculate the number of animals, the mean outcome, or the variance in each group.

### Data collection

The following items from the eligible studies were independently extracted by the two researchers (Zeng LW and Zhu YW): general study information (first author, publication year); animal species, gender and age; anesthetics used; method of ICH induction; intervention dose (initial and total dose); time of administration; route of delivery; functional outcome (neurobehavioral score measured on any scale) or pathological outcome (brain water content); number of animals per group for individual comparisons; assessment time and study quality index. For every treatment comparison (a given dose of an intervention at a given time of administration after ICH), we extracted data regarding mean and standard deviation (SD) from both the control and treatment groups to compare the drug efficacy. If data were only presented graphically, we measured values for the mean and SD from graphs using quantitative methods on highly magnified images (GetData Graph Digitizer, version 2.26). If the SD was not directly reported, we calculated it by multiplying the reported standard error (SE) by the square root of the group size. Besides, if outcome assessments were performed at different times, we only included the final time point assessment. In addition, if the study included more than one experimental group differentiated by different dosages or treatment time which was compared against a common control group, these parallel groups would be included separately as independent experiments and the control group size divided equally among the numbers of treatment groups. Moreover, if the data from multiple brain slices were reported in structural outcomes, we only extracted the data of ipsilateral basal ganglia.

### Methodological quality of studies

The quality of each experiment was assessed according to the CAMARADES checklists, which consist of the following: (1) peer-reviewed publication; (2) control of temperature; (3) random allocation to treatment or control; (4) blinded induction of hemorrhage; (5) blinded assessment of outcome; (6) use of anesthetic without marked intrinsic neuroprotective activity, such as ketamine; (7) animal model with relevant comorbidities (aged, diabetic, or hypertensive); (8) sample size calculation; (9) compliance with animal welfare regulations; and (10) statement of potential conflict of interests. We defined studies that scored < 5 points as low quality, and those that scored ≥5 points as high quality.

### Statistical analysis

The Hedges calculation was adopted to determine a comprehensive estimation of effect size with standard mean differences (SMD), and meta-analysis was performed using Stata statistical software version 12.0 (StataCorp LP, College Station, TX, USA). The effects of melatonin on the neurobehavioral score and brain water content were compared between the treatment and control groups. The percentage of heterogeneity across the studies was estimated by I^2^ statistic. An I^2^ statistic of < 25% indicated low heterogeneity, 25% to 50% indicated moderate heterogeneity, and >50% indicated high heterogeneity [44]. Fixed-effect model was used if no substantial heterogeneity was observed. On the contrary, random-effect model was used when substantial heterogeneity was observed [45]. Sensitivity analyses were performed by omitting one study at a time to evaluate whether the results were affected by a single study. Publication bias was detected by funnel plotting. Asymmetry was assessed using an Egger’s test and the trim-and-fill method [46]. Subgroup analyses were also performed to explore substantial heterogeneity using Cochrane Review Manager 5.3 (The Cochrane Collaboration). Statistical significance was set at p < 0.05, and the 95% confidence intervals (CIs) of all results were calculated.

## Supplementary Material

Supplementary Figures

Supplementary File 1
