# Supplementary material for: Efficacy of melatonin in animal models of intracerebral hemorrhage: a systematic review and meta-analysis
Source: Aging (Albany NY). 2021 Jan 27;13(2):3010–30. doi: 10.18632/aging.202457 (PMC7880339; doi:10.18632/aging.202457)
Supplement: Supplementary Figures [file aging-13-202457-s001.pdf]

SUPPLEMENTARY FIGURES

Study or Subgroup	Experimental Mean	Experimental SD	Experimental Total	Control Mean	Control SD	Control Total	Weight	Std. Mean Difference IV, Random, 95% CI
**1.14.1 positive scale**								
Lekic2011-1	5.25	1.61	8	8.96	1.33	4	6.7%	-2.24 [-3.86, -0.61]
Lekic2011-2	5.05	2.18	8	8.96	1.33	4	7.2%	-1.84 [-3.34, -0.34]
Lu2019	6.69	0.37	6	10.18	0.27	6	1.5%	-9.95 [-14.93, -4.96]
Ueda2014-1	5.79	1.76	14	7.92	1.39	12	9.5%	-1.29 [-2.15, -0.43]
Ueda2014-2	6.11	1.05	9	7.92	1.09	19	9.3%	-1.63 [-2.55, -0.71]
Wang2018	2.5	1.18	12	3.5	0.78	12	9.5%	-0.97 [-1.82, -0.11]
**Subtotal (95% CI)**			**57**			**57**	**43.8%**	**-1.74 [-2.59, -0.89]**
Heterogeneity: Tau² = 0.63; Chi² = 13.82, df = 5 (P = 0.02); I² = 64%								
Test for overall effect: Z = 4.01 (P < 0.0001)								
**1.14.2 negative scale**								
Lekic2010-1	-9.27	2.22	6	-12	4.23	6	8.3%	0.75 [-0.44, 1.93]
Lekic2010-2	-11.91	5.88	6	-11.72	4.9	3	7.6%	-0.03 [-1.42, 1.36]
Lekic2010-3	-11	6	6	-11.72	4.9	3	7.6%	0.11 [-1.28, 1.50]
Rogas2008-1	-9.3	1	4	-11.5	2	4	6.7%	1.21 [-0.41, 2.83]
Rogas2008-2	-9.8	2	4	-11.5	3.4	2	6.2%	0.56 [-1.22, 2.34]
Rogas2008-3	-11.8	1.6	4	-11.5	3.4	2	6.5%	-0.11 [-1.81, 1.59]
Xu2018-1	-14.26	1.11	6	-12.05	2	3	6.7%	-1.38 [-3.01, 0.25]
Xu2018-2	-15.36	2.08	6	-12.05	2	3	6.7%	-1.43 [-3.07, 0.21]
**Subtotal (95% CI)**			**42**			**26**	**56.2%**	**0.01 [-0.63, 0.64]**
Heterogeneity: Tau² = 0.23; Chi² = 9.76, df = 7 (P = 0.20); I² = 28%								
Test for overall effect: Z = 0.02 (P = 0.98)								
**Total (95% CI)**			**99**			**83**	**100.0%**	**-0.81 [-1.47, -0.15]**
Heterogeneity: Tau² = 0.99; Chi² = 41.48, df = 13 (P < 0.0001); I² = 69%								
Test for overall effect: Z = 2.41 (P = 0.02)								
Test for subgroup differences: Chi² = 10.43, df = 1 (P = 0.001), I² = 90.4%								

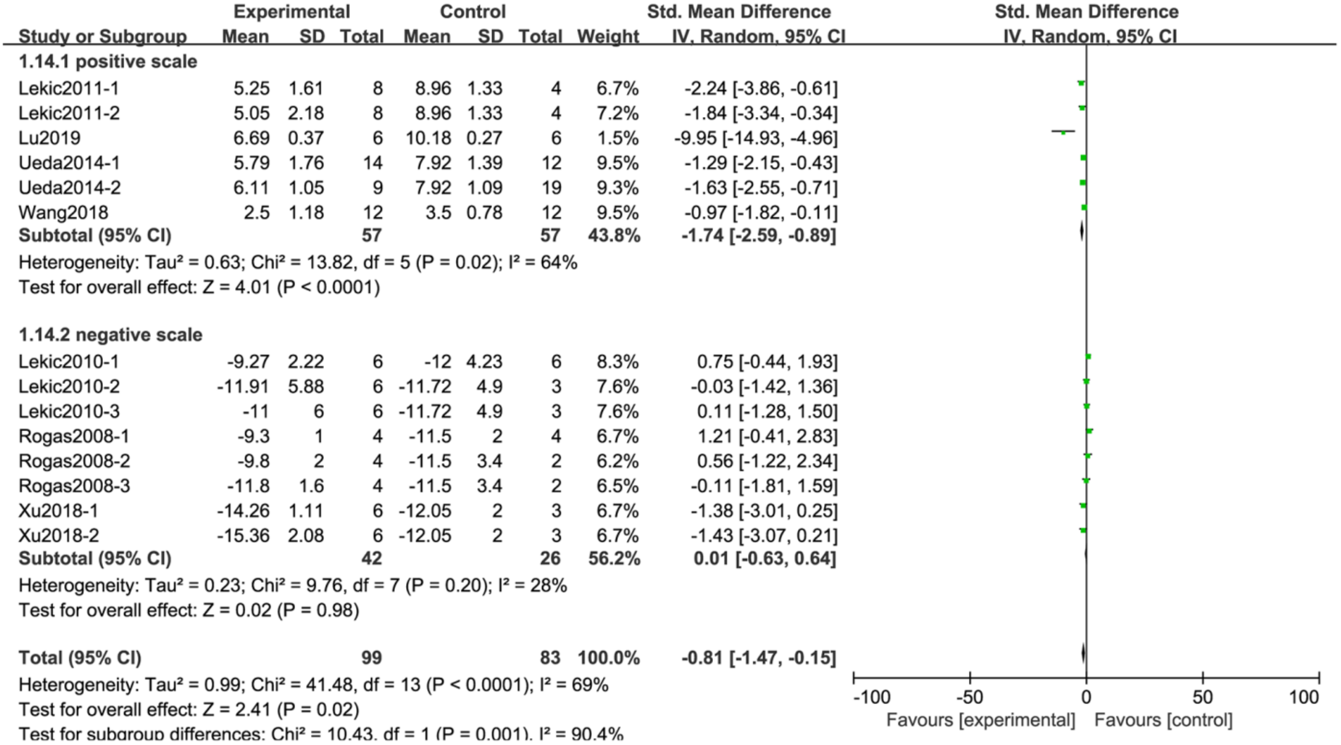

Supplementary Figure 1. Subgroup analysis by neurobehavioral scales.

Study or Subgroup	Experimental Mean	Experimental SD	Experimental Total	Control Mean	Control SD	Control Total	Weight	Std. Mean Difference IV, Random, 95% CI
**1.3.1 high quality**								
Lekic2010-1	-9.27	2.22	6	-12	4.23	6	8.3%	0.75 [-0.44, 1.93]
Lekic2010-2	-11.91	5.88	6	-11.72	4.9	3	7.6%	-0.03 [-1.42, 1.36]
Lekic2010-3	-11	6	6	-11.72	4.9	3	7.6%	0.11 [-1.28, 1.50]
Lekic2011-1	5.25	1.61	8	8.96	1.33	4	6.7%	-2.24 [-3.86, -0.61]
Lekic2011-2	5.05	2.18	8	8.96	1.33	4	7.2%	-1.84 [-3.34, -0.34]
Lu2019	6.69	0.37	6	10.18	0.27	6	1.5%	-9.95 [-14.93, -4.96]
Rogas2008-1	-9.3	1	4	-11.5	2	4	6.7%	1.21 [-0.41, 2.83]
Rogas2008-2	-9.8	2	4	-11.5	3.4	2	6.2%	0.56 [-1.22, 2.34]
Rogas2008-3	-11.8	1.6	4	-11.5	3.4	2	6.5%	-0.11 [-1.81, 1.59]
Wang2018	2.5	1.18	12	3.5	0.78	12	9.5%	-0.97 [-1.82, -0.11]
Xu2018-1	-14.26	1.11	6	-12.05	2	3	6.7%	-1.38 [-3.01, 0.25]
Xu2018-2	-15.36	2.08	6	-12.05	2	3	6.7%	-1.43 [-3.07, 0.21]
**Subtotal (95% CI)**			**76**			**52**	**81.2%**	**-0.68 [-1.48, 0.11]**
Heterogeneity: Tau² = 1.27; Chi² = 35.93, df = 11 (P = 0.0002); I² = 69%								
Test for overall effect: Z = 1.69 (P = 0.09)								
**1.3.2 low quality**								
Ueda2014-1	5.79	1.76	14	7.92	1.39	12	9.5%	-1.29 [-2.15, -0.43]
Ueda2014-2	6.11	1.05	9	7.92	1.09	19	9.3%	-1.63 [-2.55, -0.71]
**Subtotal (95% CI)**			**23**			**31**	**18.8%**	**-1.45 [-2.08, -0.82]**
Heterogeneity: Tau² = 0.00; Chi² = 0.28, df = 1 (P = 0.59); I² = 0%								
Test for overall effect: Z = 4.53 (P < 0.00001)								
**Total (95% CI)**			**99**			**83**	**100.0%**	**-0.81 [-1.47, -0.15]**
Heterogeneity: Tau² = 0.99; Chi² = 41.48, df = 13 (P < 0.0001); I² = 69%								
Test for overall effect: Z = 2.41 (P = 0.02)								
Test for subgroup differences: Chi² = 2.19, df = 1 (P = 0.14), I² = 54.4%								

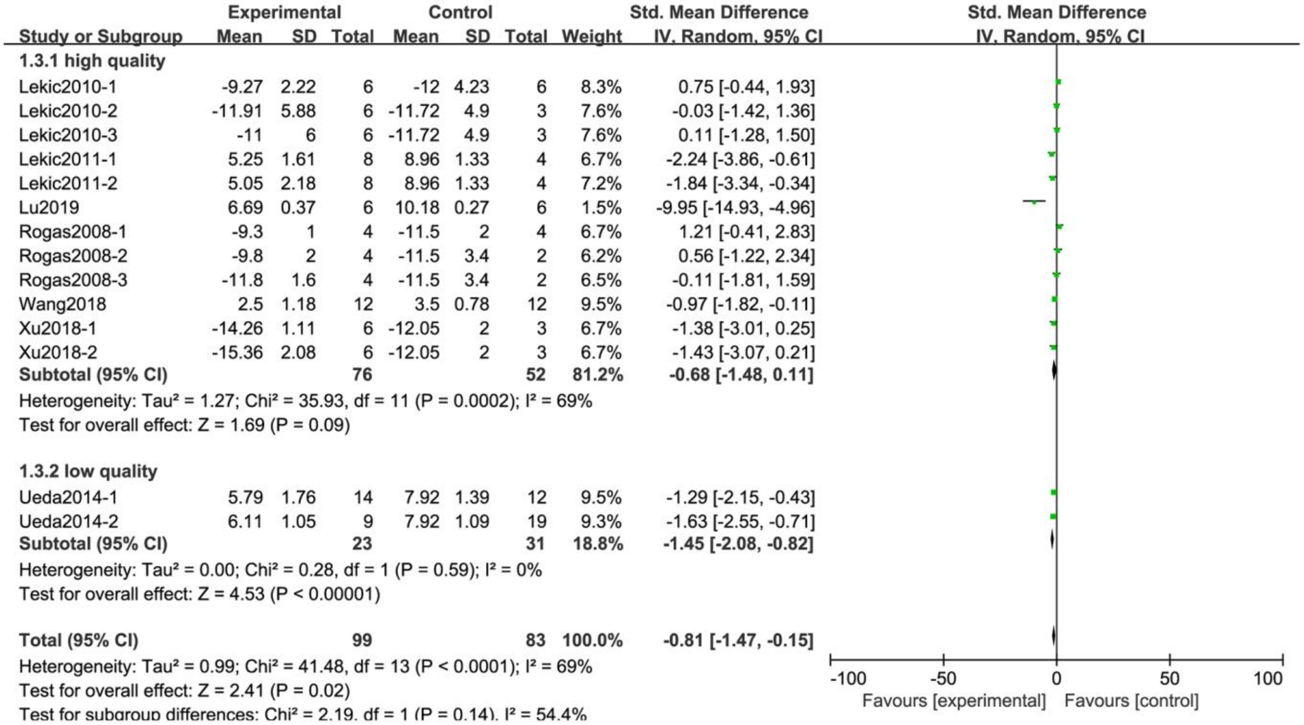

Supplementary Figure 2. Subgroup analysis by study quality.

Study or Subgroup	Experimental Mean	Experimental SD	Experimental Total	Control Mean	Control SD	Control Total	Weight	Std. Mean Difference IV, Random, 95% CI
**1.2.1 collagenase**								
Lekic2010-1	-9.27	2.22	6	-12	4.23	6	8.3%	0.75 [-0.44, 1.93]
Lekic2010-2	-11.91	5.88	6	-11.72	4.9	3	7.6%	-0.03 [-1.42, 1.36]
Lekic2010-3	-11	6	6	-11.72	4.9	3	7.6%	0.11 [-1.28, 1.50]
Lekic2011-1	5.25	1.61	8	8.96	1.33	4	6.7%	-2.24 [-3.86, -0.61]
Lekic2011-2	5.05	2.18	8	8.96	1.33	4	7.2%	-1.84 [-3.34, -0.34]
Rogas2008-1	-9.3	1	4	-11.5	2	4	6.7%	1.21 [-0.41, 2.83]
Rogas2008-2	-9.8	2	4	-11.5	3.4	2	6.2%	0.56 [-1.22, 2.34]
Rogas2008-3	-11.8	1.6	4	-11.5	3.4	2	6.5%	-0.11 [-1.81, 1.59]
Ueda2014-1	5.79	1.76	14	7.92	1.39	12	9.5%	-1.29 [-2.15, -0.43]
Ueda2014-2	6.11	1.05	9	7.92	1.09	19	9.3%	-1.63 [-2.55, -0.71]
**Subtotal (95% CI)**			**69**			**59**	**75.6%**	**-0.51 [-1.23, 0.22]**
Heterogeneity: Tau² = 0.88; Chi² = 27.05, df = 9 (P = 0.001); I² = 67%								
Test for overall effect: Z = 1.37 (P = 0.17)								
**1.2.2 whole blood**								
Lu2019	6.69	0.37	6	10.18	0.27	6	1.5%	-9.95 [-14.93, -4.96]
Wang2018	2.5	1.18	12	3.5	0.78	12	9.5%	-0.97 [-1.82, -0.11]
Xu2018-1	-14.26	1.11	6	-12.05	2	3	6.7%	-1.38 [-3.01, 0.25]
Xu2018-2	-15.36	2.08	6	-12.05	2	3	6.7%	-1.43 [-3.07, 0.21]
**Subtotal (95% CI)**			**30**			**24**	**24.4%**	**-2.03 [-3.76, -0.30]**
Heterogeneity: Tau² = 2.06; Chi² = 12.18, df = 3 (P = 0.007); I² = 75%								
Test for overall effect: Z = 2.30 (P = 0.02)								
**Total (95% CI)**			**99**			**83**	**100.0%**	**-0.81 [-1.47, -0.15]**
Heterogeneity: Tau² = 0.99; Chi² = 41.48, df = 13 (P < 0.0001); I² = 69%								
Test for overall effect: Z = 2.41 (P = 0.02)								
Test for subgroup differences: Chi² = 2.54, df = 1 (P = 0.11), I² = 60.7%								

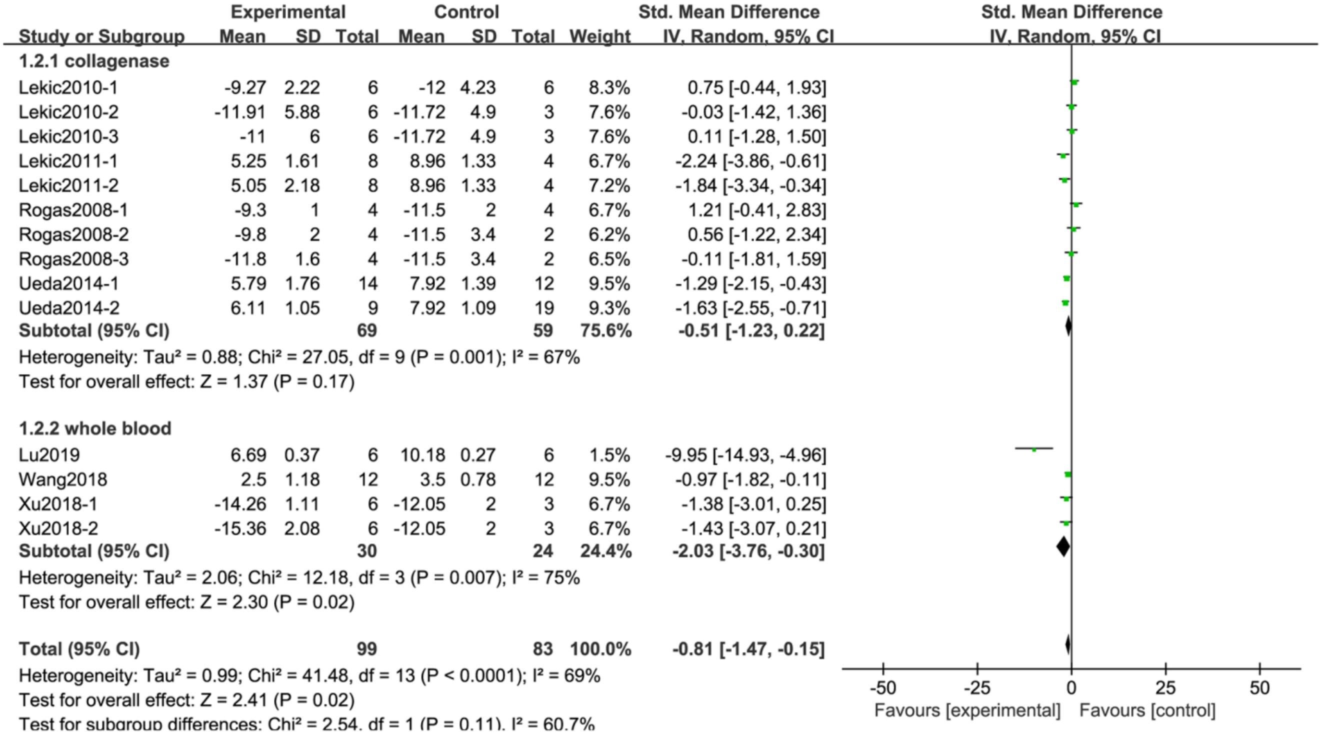

Supplementary Figure 3. Subgroup analysis by methods to induce ICH.

Study or Subgroup	Experimental Mean	Experimental SD	Experimental Total	Control Mean	Control SD	Control Total	Weight	Std. Mean Difference IV, Random, 95% CI
**1.9.1 isoflurane**								
Lekic2010-1	-9.27	2.22	6	-12	4.23	6	8.3%	0.75 [-0.44, 1.93]
Lekic2010-2	-11.91	5.88	6	-11.72	4.9	3	7.6%	-0.03 [-1.42, 1.36]
Lekic2010-3	-11	6	6	-11.72	4.9	3	7.6%	0.11 [-1.28, 1.50]
Lekic2011-1	5.25	1.61	8	8.96	1.33	4	6.7%	-2.24 [-3.86, -0.61]
Lekic2011-2	5.05	2.18	8	8.96	1.33	4	7.2%	-1.84 [-3.34, -0.34]
Rogas2008-1	-9.3	1	4	-11.5	2	4	6.7%	1.21 [-0.41, 2.83]
Rogas2008-2	-9.8	2	4	-11.5	3.4	2	6.2%	0.56 [-1.22, 2.34]
Rogas2008-3	-11.8	1.6	4	-11.5	3.4	2	6.5%	-0.11 [-1.81, 1.59]
**Subtotal (95% CI)**			**46**			**28**	**56.7%**	**-0.18 [-1.01, 0.64]**
Heterogeneity: Tau² = 0.82; Chi² = 16.90, df = 7 (P = 0.02); I² = 59%								
Test for overall effect: Z = 0.43 (P = 0.67)								
**1.9.2 pentobarbital**								
Lu2019	6.69	0.37	6	10.18	0.27	6	1.5%	-9.95 [-14.93, -4.96]
Ueda2014-1	5.79	1.76	14	7.92	1.39	12	9.5%	-1.29 [-2.15, -0.43]
Ueda2014-2	6.11	1.05	9	7.92	1.09	19	9.3%	-1.63 [-2.55, -0.71]
Xu2018-1	-14.26	1.11	6	-12.05	2	3	6.7%	-1.38 [-3.01, 0.25]
Xu2018-2	-15.36	2.08	6	-12.05	2	3	6.7%	-1.43 [-3.07, 0.21]
**Subtotal (95% CI)**			**41**			**43**	**33.7%**	**-1.78 [-2.85, -0.71]**
Heterogeneity: Tau² = 0.84; Chi² = 11.34, df = 4 (P = 0.02); I² = 65%								
Test for overall effect: Z = 3.26 (P = 0.001)								
**1.9.3 chloral hydrate**								
Wang2018	2.5	1.18	12	3.5	0.78	12	9.5%	-0.97 [-1.82, -0.11]
**Subtotal (95% CI)**			**12**			**12**	**9.5%**	**-0.97 [-1.82, -0.11]**
Heterogeneity: Not applicable								
Test for overall effect: Z = 2.22 (P = 0.03)								
**Total (95% CI)**			**99**			**83**	**100.0%**	**-0.81 [-1.47, -0.15]**
Heterogeneity: Tau² = 0.99; Chi² = 41.48, df = 13 (P < 0.0001); I² = 69%								
Test for overall effect: Z = 2.41 (P = 0.02)								
Test for subgroup differences: Chi² = 5.50, df = 2 (P = 0.06), I² = 63.7%								

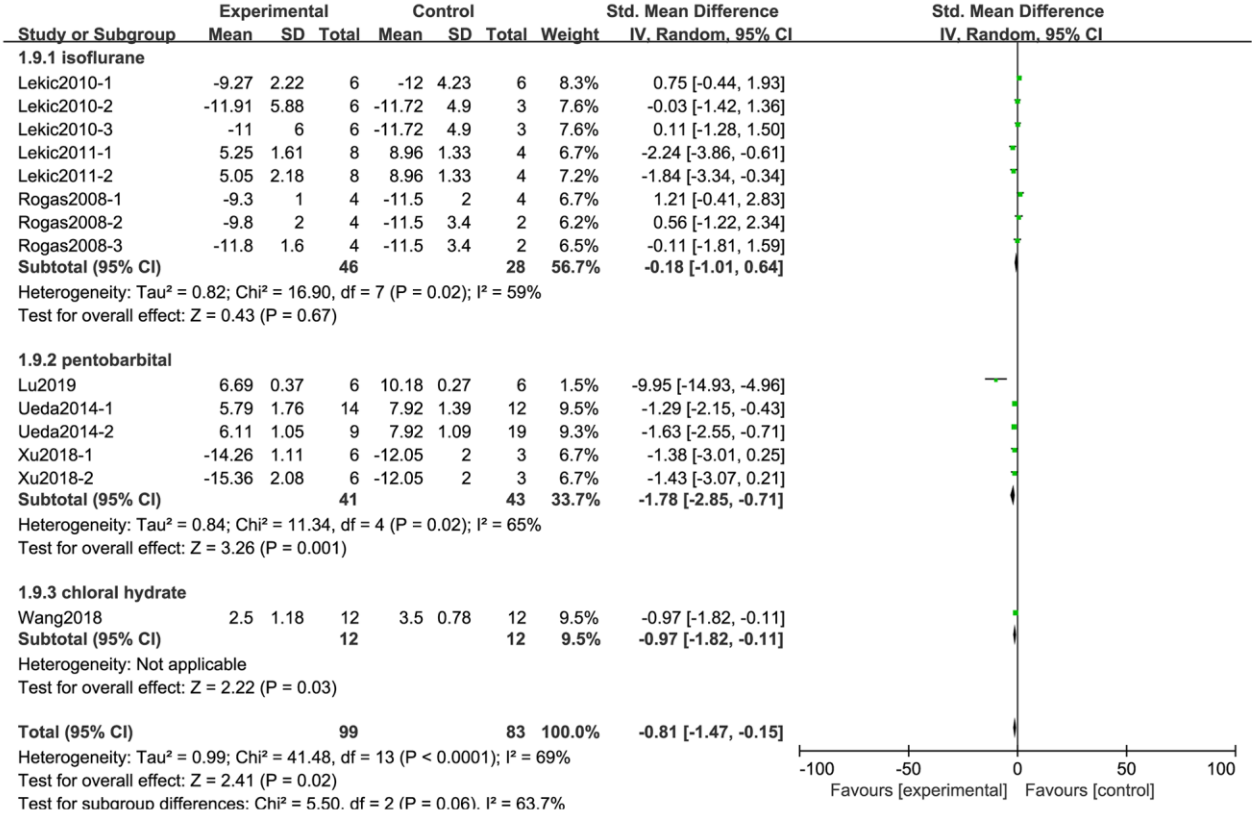

Supplementary Figure 4. Subgroup analysis by different anaesthetics.

Study or Subgroup	Experimental Mean	Experimental SD	Experimental Total	Control Mean	Control SD	Control Total	Weight	Std. Mean Difference IV, Random, 95% CI
**1.3.1 intraperitoneal injection**								
Lekic2010-1	-9.27	2.22	6	-12	4.23	6	8.3%	0.75 [-0.44, 1.93]
Lekic2010-2	-11.91	5.88	6	-11.72	4.9	3	7.6%	-0.03 [-1.42, 1.36]
Lekic2010-3	-11	6	6	-11.72	4.9	3	7.6%	0.11 [-1.28, 1.50]
Lekic2011-1	5.25	1.61	8	8.96	1.33	4	6.7%	-2.24 [-3.86, -0.61]
Lekic2011-2	5.05	2.18	8	8.96	1.33	4	7.2%	-1.84 [-3.34, -0.34]
Lu2019	6.69	0.37	6	10.18	0.27	6	1.5%	-9.95 [-14.93, -4.96]
Rogas2008-1	-9.3	1	4	-11.5	2	4	6.7%	1.21 [-0.41, 2.83]
Rogas2008-2	-9.8	2	4	-11.5	3.4	2	6.2%	0.56 [-1.22, 2.34]
Rogas2008-3	-11.8	1.6	4	-11.5	3.4	2	6.5%	-0.11 [-1.81, 1.59]
Wang2018	2.5	1.18	12	3.5	0.78	12	9.5%	-0.97 [-1.82, -0.11]
Xu2018-1	-14.26	1.11	6	-12.05	2	3	6.7%	-1.38 [-3.01, 0.25]
Xu2018-2	-15.36	2.08	6	-12.05	2	3	6.7%	-1.43 [-3.07, 0.21]
**Subtotal (95% CI)**			**76**			**52**	**81.2%**	**-0.68 [-1.48, 0.11]**
Heterogeneity: Tau² = 1.27; Chi² = 35.93, df = 11 (P = 0.0002); I² = 69%								
Test for overall effect: Z = 1.69 (P = 0.09)								
**1.3.2 oral administration**								
Ueda2014-1	5.79	1.76	14	7.92	1.39	12	9.5%	-1.29 [-2.15, -0.43]
Ueda2014-2	6.11	1.05	9	7.92	1.09	19	9.3%	-1.63 [-2.55, -0.71]
**Subtotal (95% CI)**			**23**			**31**	**18.8%**	**-1.45 [-2.08, -0.82]**
Heterogeneity: Tau² = 0.00; Chi² = 0.28, df = 1 (P = 0.59); I² = 0%								
Test for overall effect: Z = 4.53 (P < 0.00001)								
**Total (95% CI)**			**99**			**83**	**100.0%**	**-0.81 [-1.47, -0.15]**
Heterogeneity: Tau² = 0.99; Chi² = 41.48, df = 13 (P < 0.0001); I² = 69%								
Test for overall effect: Z = 2.41 (P = 0.02)								
Test for subgroup differences: Chi² = 2.19, df = 1 (P = 0.14), I² = 54.4%								

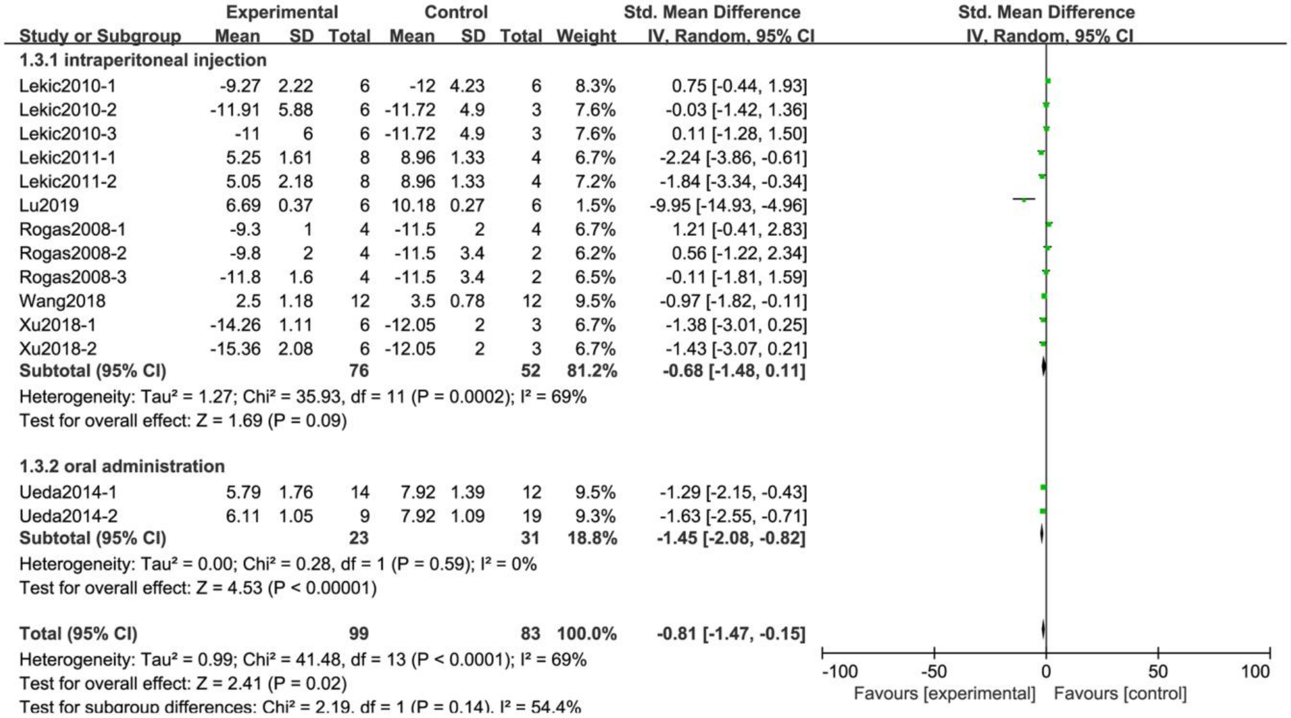

Supplementary Figure 5. Subgroup analysis by routes of injection.

Study or Subgroup	Experimental Mean	Experimental SD	Experimental Total	Control Mean	Control SD	Control Total	Weight	Std. Mean Difference IV, Random, 95% CI
**1.13.1 5mg/kg**								
Lekic2010-2	-11.91	5.88	6	-11.72	4.9	3	7.6%	-0.03 [-1.42, 1.36]
Lekic2011-1	5.25	1.61	8	8.96	1.33	4	6.7%	-2.24 [-3.86, -0.61]
**Subtotal (95% CI)**			**14**			**7**	**14.3%**	**-1.09 [-3.25, 1.07]**
Heterogeneity: Tau² = 1.84; Chi² = 4.11, df = 1 (P = 0.04); I² = 76%								
Test for overall effect: Z = 0.99 (P = 0.32)								
**1.13.2 10mg/kg**								
Lekic2011-2	5.05	2.18	8	8.96	1.33	4	7.2%	-1.84 [-3.34, -0.34]
**Subtotal (95% CI)**			**8**			**4**	**7.2%**	**-1.84 [-3.34, -0.34]**
Heterogeneity: Not applicable								
Test for overall effect: Z = 2.40 (P = 0.02)								
**1.13.3 15mg/kg**								
Lekic2010-1	-9.27	2.22	6	-12	4.23	6	8.3%	0.75 [-0.44, 1.93]
Lekic2010-3	-11	6	6	-11.72	4.9	3	7.6%	0.11 [-1.28, 1.50]
Rogas2008-1	-9.3	1	4	-11.5	2	4	6.7%	1.21 [-0.41, 2.83]
Rogas2008-2	-9.8	2	4	-11.5	3.4	2	6.2%	0.56 [-1.22, 2.34]
Wang2018	2.5	1.18	12	3.5	0.78	12	9.5%	-0.97 [-1.82, -0.11]
**Subtotal (95% CI)**			**32**			**27**	**38.4%**	**0.20 [-0.67, 1.08]**
Heterogeneity: Tau² = 0.53; Chi² = 8.97, df = 4 (P = 0.06); I² = 55%								
Test for overall effect: Z = 0.45 (P = 0.65)								
**1.13.4 20mg/kg**								
Lu2019	6.69	0.37	6	10.18	0.27	6	1.5%	-9.95 [-14.93, -4.96]
**Subtotal (95% CI)**			**6**			**6**	**1.5%**	**-9.95 [-14.93, -4.96]**
Heterogeneity: Not applicable								
Test for overall effect: Z = 3.91 (P < 0.0001)								
**1.13.5 100mg/kg**								
Xu2018-1	-14.26	1.11	6	-12.05	2	3	6.7%	-1.38 [-3.01, 0.25]
**Subtotal (95% CI)**			**6**			**3**	**6.7%**	**-1.38 [-3.01, 0.25]**
Heterogeneity: Not applicable								
Test for overall effect: Z = 1.66 (P = 0.10)								
**1.13.6 105mg/kg**								
Ueda2014-1	5.79	1.76	14	7.92	1.39	12	9.5%	-1.29 [-2.15, -0.43]
Ueda2014-2	6.11	1.05	9	7.92	1.09	19	9.3%	-1.63 [-2.55, -0.71]
**Subtotal (95% CI)**			**23**			**31**	**18.8%**	**-1.45 [-2.08, -0.82]**
Heterogeneity: Tau² = 0.00; Chi² = 0.28, df = 1 (P = 0.59); I² = 0%								
Test for overall effect: Z = 4.53 (P < 0.00001)								
**1.13.7 150mg/kg**								
Rogas2008-3	-11.8	1.6	4	-11.5	3.4	2	6.5%	-0.11 [-1.81, 1.59]
Xu2018-2	-15.36	2.08	6	-12.05	2	3	6.7%	-1.43 [-3.07, 0.21]
**Subtotal (95% CI)**			**10**			**5**	**13.1%**	**-0.79 [-2.08, 0.50]**
Heterogeneity: Tau² = 0.14; Chi² = 1.20, df = 1 (P = 0.27); I² = 17%								
Test for overall effect: Z = 1.20 (P = 0.23)								
**Total (95% CI)**			**99**			**83**	**100.0%**	**-0.81 [-1.47, -0.15]**
Heterogeneity: Tau² = 0.99; Chi² = 41.48, df = 13 (P < 0.0001); I² = 69%								
Test for overall effect: Z = 2.41 (P = 0.02)								
Test for subgroup differences: Chi² = 23.01, df = 6 (P = 0.0008), I² = 73.9%								

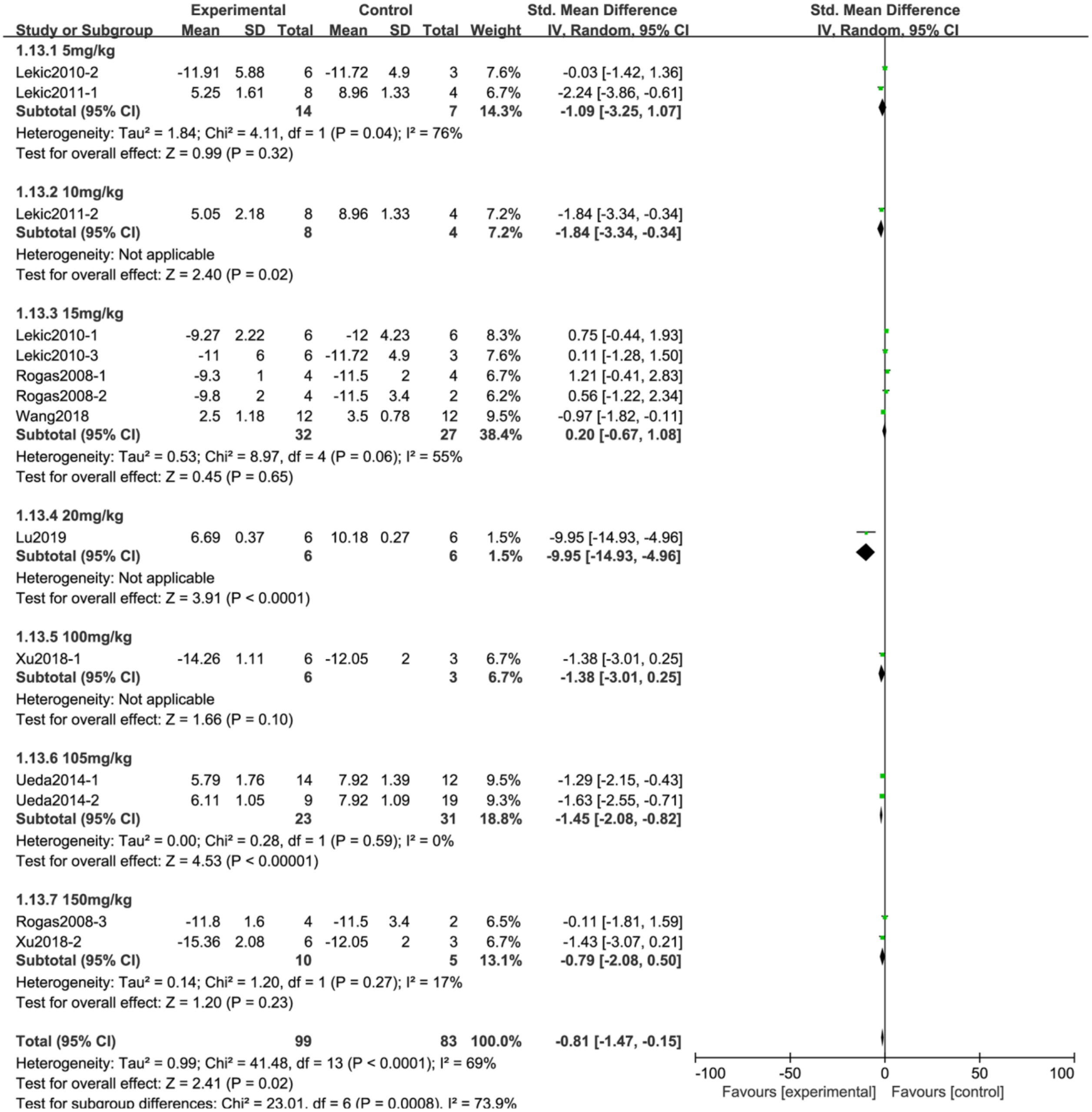

Supplementary Figure 6. Subgroup analysis by different dosages.

Study or Subgroup	Experimental Mean	Experimental SD	Experimental Total	Control Mean	Control SD	Control Total	Weight	Std. Mean Difference IV, Random, 95% CI
**1.4.1 single dose**								
Lekic2010-1	-9.27	2.22	6	-12	4.23	6	8.3%	0.75 [-0.44, 1.93]
Lekic2010-2	-11.91	5.88	6	-11.72	4.9	3	7.6%	-0.03 [-1.42, 1.36]
Lekic2010-3	-11	6	6	-11.72	4.9	3	7.6%	0.11 [-1.28, 1.50]
Lekic2011-1	5.25	1.61	8	8.96	1.33	4	6.7%	-2.24 [-3.86, -0.61]
Lekic2011-2	5.05	2.18	8	8.96	1.33	4	7.2%	-1.84 [-3.34, -0.34]
Lu2019	6.69	0.37	6	10.18	0.27	6	1.5%	-9.95 [-14.93, -4.96]
Rogas2008-1	-9.3	1	4	-11.5	2	4	6.7%	1.21 [-0.41, 2.83]
Rogas2008-2	-9.8	2	4	-11.5	3.4	2	6.2%	0.56 [-1.22, 2.34]
Rogas2008-3	-11.8	1.6	4	-11.5	3.4	2	6.5%	-0.11 [-1.81, 1.59]
Xu2018-1	-14.26	1.11	6	-12.05	2	3	6.7%	-1.38 [-3.01, 0.25]
Xu2018-2	-15.36	2.08	6	-12.05	2	3	6.7%	-1.43 [-3.07, 0.21]
**Subtotal (95% CI)**			**64**			**40**	**71.6%**	**-0.68 [-1.60, 0.24]**
Heterogeneity: Tau² = 1.64; Chi² = 34.83, df = 10 (P = 0.0001); I² = 71%								
Test for overall effect: Z = 1.46 (P = 0.15)								
**1.4.2 multiple dosage**								
Ueda2014-1	5.79	1.76	14	7.92	1.39	12	9.5%	-1.29 [-2.15, -0.43]
Ueda2014-2	6.11	1.05	9	7.92	1.09	19	9.3%	-1.63 [-2.55, -0.71]
Wang2018	2.5	1.18	12	3.5	0.78	12	9.5%	-0.97 [-1.82, -0.11]
**Subtotal (95% CI)**			**35**			**43**	**28.4%**	**-1.28 [-1.78, -0.77]**
Heterogeneity: Tau² = 0.00; Chi² = 1.08, df = 2 (P = 0.58); I² = 0%								
Test for overall effect: Z = 4.96 (P < 0.00001)								
**Total (95% CI)**			**99**			**83**	**100.0%**	**-0.81 [-1.47, -0.15]**
Heterogeneity: Tau² = 0.99; Chi² = 41.48, df = 13 (P < 0.0001); I² = 69%								
Test for overall effect: Z = 2.41 (P = 0.02)								
Test for subgroup differences: Chi² = 1.24, df = 1 (P = 0.27), I² = 19.4%								

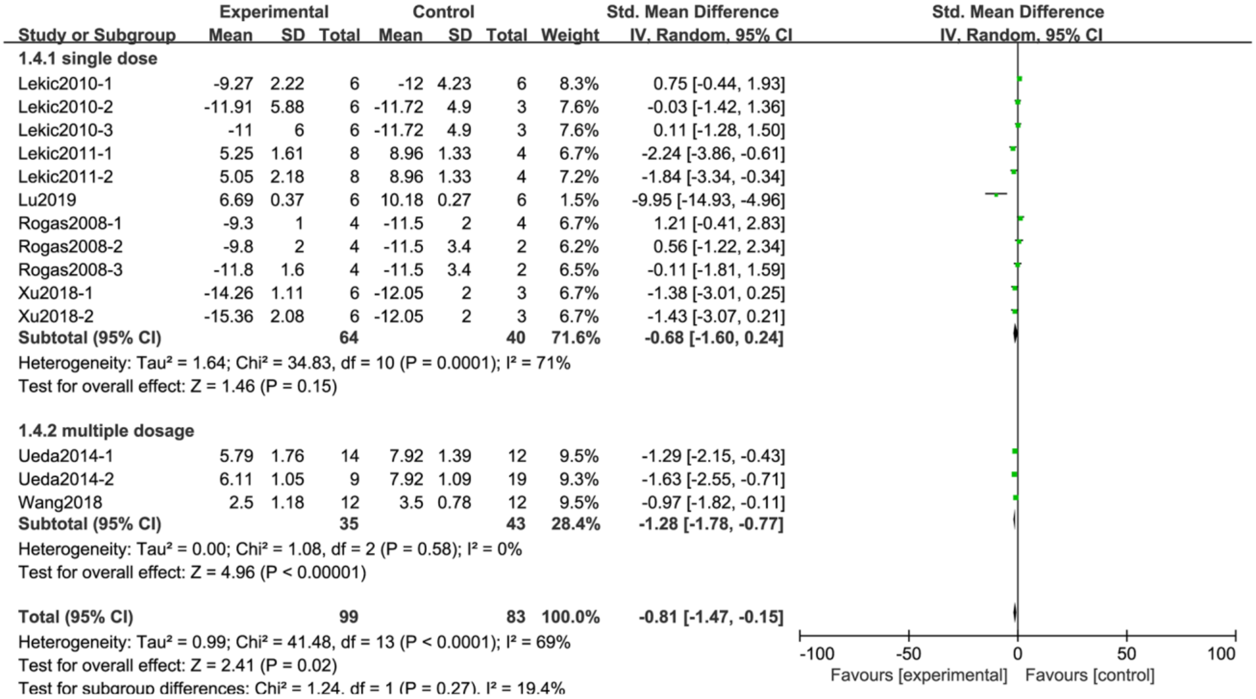

Supplementary Figure 7. Subgroup analysis by times of melatonin administration.

Study or Subgroup	Experimental Mean	Experimental SD	Experimental Total	Control Mean	Control SD	Control Total	Weight	Std. Mean Difference IV, Random, 95% CI
**1.6.1 pre group**								
Lu2019	6.69	0.37	6	10.18	0.27	6	1.5%	-9.95 [-14.93, -4.96]
Ueda2014-2	6.11	1.05	9	7.92	1.09	19	9.3%	-1.63 [-2.55, -0.71]
**Subtotal (95% CI)**			**15**			**25**	**10.8%**	**-5.41 [-13.53, 2.70]**
Heterogeneity: Tau² = 31.24; Chi² = 10.34, df = 1 (P = 0.001); I² = 90%								
Test for overall effect: Z = 1.31 (P = 0.19)								
**1.6.2 post**								
Lekic2010-1	-9.27	2.22	6	-12	4.23	6	8.3%	0.75 [-0.44, 1.93]
Lekic2010-2	-11.91	5.88	6	-11.72	4.9	3	7.6%	-0.03 [-1.42, 1.36]
Lekic2010-3	-11	6	6	-11.72	4.9	3	7.6%	0.11 [-1.28, 1.50]
Lekic2011-1	5.25	1.61	8	8.96	1.33	4	6.7%	-2.24 [-3.86, -0.61]
Lekic2011-2	5.05	2.18	8	8.96	1.33	4	7.2%	-1.84 [-3.34, -0.34]
Rogas2008-1	-9.3	1	4	-11.5	2	4	6.7%	1.21 [-0.41, 2.83]
Rogas2008-2	-9.8	2	4	-11.5	3.4	2	6.2%	0.56 [-1.22, 2.34]
Rogas2008-3	-11.8	1.6	4	-11.5	3.4	2	6.5%	-0.11 [-1.81, 1.59]
Ueda2014-1	5.79	1.76	14	7.92	1.39	12	9.5%	-1.29 [-2.15, -0.43]
Wang2018	2.5	1.18	12	3.5	0.78	12	9.5%	-0.97 [-1.82, -0.11]
Xu2018-1	-14.26	1.11	6	-12.05	2	3	6.7%	-1.38 [-3.01, 0.25]
Xu2018-2	-15.36	2.08	6	-12.05	2	3	6.7%	-1.43 [-3.07, 0.21]
**Subtotal (95% CI)**			**84**			**58**	**89.2%**	**-0.58 [-1.17, 0.01]**
Heterogeneity: Tau² = 0.57; Chi² = 24.84, df = 11 (P = 0.010); I² = 56%								
Test for overall effect: Z = 1.92 (P = 0.06)								
**Total (95% CI)**			**99**			**83**	**100.0%**	**-0.81 [-1.47, -0.15]**
Heterogeneity: Tau² = 0.99; Chi² = 41.48, df = 13 (P < 0.0001); I² = 69%								
Test for overall effect: Z = 2.41 (P = 0.02)								
Test for subgroup differences: Chi² = 1.36, df = 1 (P = 0.24), I² = 26.3%								

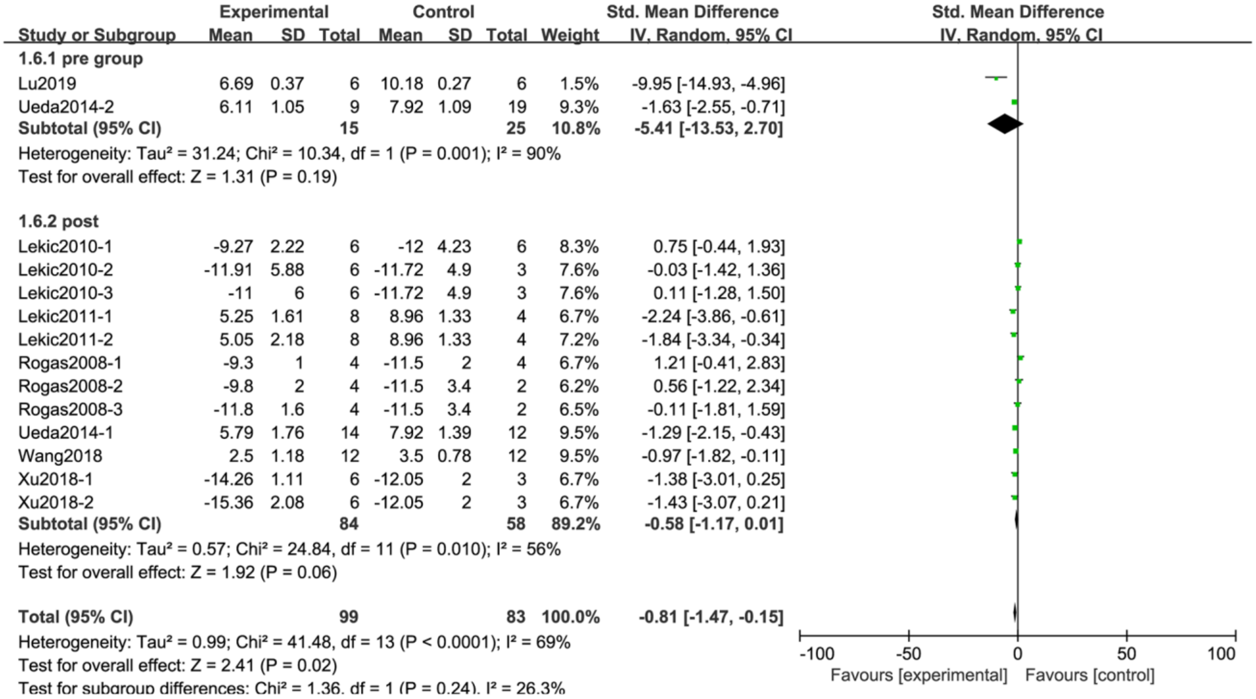

Supplementary Figure 8. Subgroup analysis by time administration.
