# Supplementary material for: Efficacy of melatonin in animal models of intracerebral hemorrhage: a systematic review and meta-analysis
Source: Aging (Albany NY). 2021 Jan 27;13(2):3010–30. doi: 10.18632/aging.202457 (PMC7880339; doi:10.18632/aging.202457)
Supplement: Supplementary File 1 [file aging-13-202457-s002.docx]

Pubmed：

((((melatonin receptor agonist[Title/Abstract]) OR (5-methoxytryptamine[Title/Abstract])) OR (melatonin[Title/Abstract] OR melatonergic agent[Title/Abstract])) OR (melatonin[MeSH Major Topic])) AND ((Cerebral Hemorrhage[MeSH Major Topic]) OR ((((((((((((((((((((((Hemorrhage, Cerebrum[Title/Abstract]) OR (Cerebrum Hemorrhage[Title/Abstract])) OR (Hemorrhages, Cerebrum[Title/Abstract])) OR (Cerebral Parenchymal Hemorrhage[Title/Abstract])) OR (Cerebral Parenchymal Hemorrhages[Title/Abstract])) OR (Hemorrhage, Cerebral Parenchymal[Title/Abstract])) OR (Hemorrhages, Cerebral Parenchymal[Title/Abstract])) OR (Parenchymal Hemorrhage, Cerebral[Title/Abstract])) OR (Parenchymal Hemorrhages, Cerebral[Title/Abstract])) OR (Intracerebral Hemorrhage[Title/Abstract])) OR (Hemorrhage, Intracerebral[Title/Abstract])) OR (Hemorrhages, Intracerebral[Title/Abstract])) OR (Intracerebral Hemorrhages[Title/Abstract])) OR (Hemorrhage, Cerebral[Title/Abstract])) OR (Cerebral Hemorrhages[Title/Abstract])) OR (Hemorrhages, Cerebral[Title/Abstract])) OR (Brain Hemorrhage, Cerebral[Title/Abstract])) OR (Brain Hemorrhages, Cerebral[Title/Abstract])) OR (Cerebral Brain Hemorrhage[Title/Abstract])) OR (Cerebral Brain Hemorrhages[Title/Abstract])) OR (Hemorrhage, Cerebral Brain[Title/Abstract])) OR (Hemorrhages, Cerebral Brain[Title/Abstract]))) = 22

Web of science：

#1 TS= (Cerebral Hemorrhage OR Hemorrhage, Cerebrum OR Cerebrum Hemorrhage OR Hemorrhages, Cerebrum OR Cerebral Parenchymal Hemorrhage OR Cerebral Parenchymal Hemorrhages OR Hemorrhage, Cerebral Parenchymal OR Hemorrhages, Cerebral Parenchymal OR Parenchymal Hemorrhage, Cerebral OR Parenchymal Hemorrhages, Cerebral OR Intracerebral Hemorrhage OR Hemorrhage, Intracerebral OR Hemorrhages, Intracerebral OR Intracerebral Hemorrhages OR Hemorrhage, Cerebral OR Cerebral Hemorrhages OR Hemorrhages, Cerebral OR Brain Hemorrhage, Cerebral OR Brain Hemorrhages, Cerebral OR Cerebral Brain Hemorrhage OR Cerebral Brain Hemorrhages OR Hemorrhage, Cerebral Brain OR Hemorrhages, Cerebral Brain)

#2 TS=(melatonin OR melatonin receptor agonist OR 5-methoxytryptamine OR melatonergic agent)

#3=#1 AND #2, = 98

EMBASE:

#1 'melatonin'/exp OR 'melatonin receptor agonist':ab,ti OR '5- methoxytryptamine':ab,ti OR 'melatonergic agent':ab,ti

#2 'brain hemorrhage'/exp OR 'hemorrhage, cerebrum':ab,ti OR 'cerebrum hemorrhage':ab,ti OR 'cerebrum hemorrhages':ab,ti OR 'hemorrhages, cerebrum':ab,ti OR 'cerebral parenchymal hemorrhage':ab,ti OR 'cerebral parenchymal hemorrhages':ab,ti OR 'hemorrhage, cerebral parenchymal':ab,ti OR 'hemorrhages, cerebral parenchymal':ab,ti OR 'parenchymal hemorrhage, cerebral':ab,ti OR 'parenchymal hemorrhages, cerebral':ab,ti OR 'intracerebral hemorrhage':ab,ti OR 'hemorrhage, intracerebral':ab,ti OR 'hemorrhages, intracerebral':ab,ti OR 'intracerebral hemorrhages':ab,ti OR 'hemorrhage, cerebral':ab,ti OR 'cerebral hemorrhages':ab,ti OR 'hemorrhages, cerebral':ab,ti OR 'brain hemorrhage, cerebral':ab,ti OR 'brain hemorrhages, cerebral':ab,ti OR 'cerebral brain hemorrhage':ab,ti OR 'cerebral brain hemorrhages':ab,ti OR 'hemorrhage, cerebral brain':ab,ti OR 'hemorrhages, cerebral brain':ab,ti

#3=#1 AND #2

（162篇）
